# Clinical significance of self-reported cough intensity and frequency in patients with interstitial lung disease: a cross-sectional study

**DOI:** 10.1186/s12890-019-1012-6

**Published:** 2019-12-16

**Authors:** Ryuhei Sato, Tomohiro Handa, Hisako Matsumoto, Takeshi Kubo, Toyohiro Hirai

**Affiliations:** 1 Department of Critical Care Nursing, Graduate School of Medicine, Kyoto University, 53 Shogoin Kawahara-cho, Sakyo-ku, Kyoto, 606-8507 Japan; 2 Department of Advanced Medicine for Respiratory Failure, Graduate School of Medicine, Kyoto University, 54 Shogoin Kawahara-cho, Sakyo-ku, Kyoto, 606-8507 Japan; 3 Department of Respiratory Medicine, Graduate School of Medicine, Kyoto University, 54 Shogoin Kawahara-cho, Sakyo-ku, Kyoto, 606-8507 Japan; 4 Department of Diagnostic Imaging and Nuclear Medicine, Graduate School of Medicine, Kyoto University, 54 Shogoin Kawahara-cho, Sakyo-ku, Kyoto, 606-8507 Japan

**Keywords:** Cough frequency, Cough intensity, Interstitial lung disease

## Abstract

**Background:**

The intensity and frequency of cough remain unclear in interstitial lung disease (ILD). The aim of this study was to evaluate the intensity and frequency of cough in idiopathic interstitial pneumonias (IIPs), connective tissue disease-associated interstitial lung disease (CTD-ILD), and chronic hypersensitivity pneumonia (CHP), and examine their associations with clinical indices.

**Methods:**

In this cross-sectional study, the intensity and frequency of cough were evaluated using a 100-mm visual analogue scale. Scores on the Leicester Cough Questionnaire, chronic dyspnoea scale, and a frequency scale for symptoms of gastro-oesophageal reflux disease (FSSG) were collected. The correlations of cough intensity and frequency with potential predictor variables were tested using bivariate and multiple logistic regression analysis.

**Results:**

The study included 70 patients with IIPs, 49 with CTD-ILD, and 10 with CHP. Patients with IIPs had the most severe cough intensity among the three patient groups. In patients with IIPs, both the intensity and frequency of cough were negatively associated with the diffusing capacity of the lung for carbon monoxide and positively with the Composite Physiologic Index (CPI). In CTD-ILD, both the intensity and frequency of cough were correlated with a higher FSSG score. In multivariate analysis of patients with ILD, IIPs and the FSSG score were independently associated with both components of cough, and CPI tended to be independently associated with cough frequency. Finally, we examined the features of the differences between cough intensity and frequency in all patients with ILD. Patients in whom cough frequency was predominant had a greater impairment of health status relative to other patients.

**Conclusions:**

Cough intensity was greater in IIPs than in other ILDs. Different clinical indices were associated with patient-reported cough intensity and frequency according to the subtype of ILD. Cough frequency was more strongly associated with health status than was cough intensity. These findings suggest that medical staff could manage patients with ILD by considering cough-related factors when assessing the intensity and frequency of cough.

## Background

Nonproductive cough is a prominent symptom of interstitial lung disease (ILD) [1], and it has been suggested that cough associated with ILD should be assessed in clinical trials [2]. The prevalence of cough has been reported to be as high as 84% in patients with idiopathic pulmonary fibrosis (IPF) [3], 73% in those with scleroderma-related ILD [4], and 83% in those with chronic hypersensitivity pneumonitis (CHP) [5]. Although cough is an important defense mechanism that serves to remove foreign material from the airways, chronic cough is associated with the impairment of health-related quality of life (HRQoL) [6, 7]. Several studies in patients with ILD have shown associations of objective measurements of cough frequency and subjective visual analogue scale (VAS) cough scores with HRQoL [5, 8].

In addition to the impairment of HRQoL, cough in patients with ILD may correlate with either disease progression or augment the activation of profibrotic mechanisms [9, 10]. Ryerson et al. reported that cough in patients with IPF was an independent predictor of disease progression and might predict time until death or need for lung transplantation [3]. Moreover, Theodore et al. and Tashkin et al. found that an increasing frequency of cough in patients with scleroderma-related ILD was correlated with the extent of fibrosis observed on high-resolution computed tomography (HRCT) images [4, 11].

The Leicester Cough Questionnaire (LCQ) [8] and cough frequency monitoring [12] are both used to assess cough in patients with IPF, in particular its frequency. However, Froese et al. confirmed that both the intensity and frequency of mechanical stress-related breathing impacted on the activation of key fibrogenic mediators, particularly transforming growth factor beta-1, in rat fibrotic tissue [13]. This suggests that it is important to evaluate cough intensity as well as its frequency in patients with ILD. However, there is a paucity of data focusing on these two components of cough separately in patients with ILD, and their correlations with baseline clinical indices representing disease severity.

The aims of this study were to evaluate both the intensity and frequency of cough in patients with ILD, and to examine their association with clinical indices that include disease severity data.

## Methods

### Study design and population

To minimise selection bias, consecutive new patients with ILD, i.e., idiopathic interstitial pneumonias (IIPs), connective tissue disease-associated interstitial lung disease (CTD-ILD), or CHP were invited to participate in this cross-sectional study. The study was performed at Kyoto University Hospital between August 2015 and March 2018. IIPs were diagnosed as previously described [14–16]. IPF was diagnosed by either surgical lung biopsy or HRCT according to the guidelines [17]. CHP was diagnosed in multidisciplinary team discussions according to the established criteria [18]. Patients who had had a respiratory tract infection in the preceding month, those who had lung cancer, postnasal drip, rhinitis, catarrhal symptoms, or a history of adult asthma, and those younger than 20 years of age were excluded.

### Measurements

Data were obtained from patient-completed questionnaires and electronic patient records. The intensity and frequency of cough were evaluated using a 100-mm VAS (0, no cough; 100, unbearable), which is the most commonly used tool to subjectively assess cough severity [19]. The VAS was used in the standardised manner recommended by the CHEST Expert Cough Panel in 2015 [20]. Patients were asked to place a vertical mark on the scale reflecting the intensity of their cough, and another vertical mark indicating the frequency of their cough. To explore the features underlying any difference between the VAS scores for cough intensity and cough frequency, we stratified patients into three categories using the following formula: (cough intensity) − (cough frequency), defining a cough frequency-dominant group, ≤ − 10 mm; an equal cough severity group, − 9 mm to 9 mm; and a cough intensity-dominant group, ≥10 mm. We assessed cough-specific HRQoL using the Japanese version of the LCQ [21]. The total score on the LCQ ranges from 3 to 21, with lower scores indicating greater impairment of health status as a result of cough. The LCQ has been previously shown to have appropriate feasibility and sensitivity; thus, it can be considered an appropriate clinical outcome tool for use in clinical trials that include patients with ILD [22]. Permission to use the LCQ was obtained from the developer (Dr Surinder Birring) and translators (Drs Akio Niimi and Haruhiko Ogawa). Dyspnoea was assessed using the Medical Research Council (MRC) chronic dyspnoea scale. The symptoms of gastro-oesophageal reflux disease (GORD) were assessed using the frequency scale for symptoms of GORD (FSSG) developed by Kusano et al. [23], which has been previously used in studies of cough [24, 25]. This questionnaire consists of 12 questions; the total score on the FSSG ranges from 0 to 48, with higher scores indicating more severe symptoms of GORD. We simultaneously collected data on the intensity and frequency of cough, LCQ, MRC chronic dyspnea scale, and FSSG scores. Additional data, including for age, sex, body mass index, smoking history, anamnesis, medication, and pulmonary function tests performed within 3 months of the assessment of cough intensity and frequency, were obtained from the clinical records. The pulmonary function tests were performed using the CHESTAC system (Chest M.I. Inc., Tokyo, Japan), and the diffusing capacity of the lung for carbon monoxide (D_Lco_) was determined by the single-breath technique. The Composite Physiologic Index (CPI) was used to predict the extent of fibrosis on HRCT. The formula used to calculate the CPI is as follows: CPI = 91.0 − (0.65 × percent predicted D_LCO_) − (0.53 × percent predicted forced vital capacity [FVC]) + (0.34 × percentage of predicted forced expiratory volume in 1 s [FEV_1_]) [26].

### Statistical analysis

The sample size was determined by the correlation between the severity of cough and %FVC in patients with IPF based on previous data [3]. However, there were few reports available on the correlation between severity of cough and %FVC in patients with ILD until the present study. Therefore, we hypothesised a mild effect size of 0.3 with an alpha level of 5% and a power of 90% and calculated that a minimum sample size of 113 would be required for this study.

The data were compared between the three groups using either the chi-square test or Fisher’s exact test for categorical variables, and either one-way analysis of variance or the Kruskal–Wallis test for continuous variables. If the one-way analysis of variance or Kruskal–Wallis test result was significant, the three groups were compared using either Tukey’s test or a Bonferroni-corrected significance level of 1.6%. The correlations of cough intensity and frequency with potential predictor variables were tested using either the Mann–Whitney *U* test or Spearman’s rank correlation coefficient as appropriate. After the cough intensity and frequency data were converted to a dichotomous variable using the median value, multiple logistic regression analysis was performed to identify independent variables predicting cough intensity and frequency. Only one variable in a set of variables with a correlation coefficient > 0.5 was used in the multivariate logistic regression analysis because of multicollinearity. Potential predictive variables were age, sex, pack-years of smoking, body mass index, FSSG, MRC chronic dyspnoea scale and CPI scores, use of glucocorticoid medication, antifibrotic therapy and proton pump inhibitors, and type of ILD. Variables were selected using a forward selection method based on the likelihood ratio test. The goodness of fit of the model was assessed using the Hosmer–Lemeshow test. Missing data were not included in the analyses. The data are expressed as the number and percentage, mean and standard deviation, or median with the interquartile range. All statistical analyses were performed using SPSS software (version 25; IBM Corp., Armonk, NY, USA). A *p*-value < 0.05 for a two-tailed test was considered statistically significant.

## Results

### Patient characteristics and intensity and frequency of cough

Twenty-one of 150 patients examined for eligibility were excluded (9 with a respiratory tract infection in the preceding month, 1 with lung cancer, 6 with rhinitis, 3 with a history of adult asthma, 1 with an acute exacerbation of IPF in the previous month, and 1 with pneumoconiosis). Seventy patients with IIPs, 49 with CTD-ILD, and 10 with CHP were included in the study. The 70 patients with IIPs included 36 (51.4%) with IPF, 2 (2.9%) with idiopathic nonspecific interstitial pneumonia, 2 (2.9%) with respiratory bronchiolitis-associated ILD, 1 (1.4%) with desquamative interstitial pneumonia, 2 (2.9%) with cryptogenic organising pneumonia, 5 (7.1%) with idiopathic pleuroparenchymal fibroelastosis, and 22 (31.4%) with unclassifiable idiopathic interstitial pneumonia. The 49 patients with CTD-ILD included 15 (30.6%) with systemic sclerosis (SSc), 10 (20.4%) with polymyositis or dermatomyositis, 8 (16.3%) with Sjögren’s syndrome, 11 (22.4%) with rheumatoid arthritis, 1 (2.0%) with systemic lupus erythematosus, and 4 (8.2%) with microscopic polyangiitis. None of the patients were using angiotensin-converting enzyme inhibitors.

Table 1 shows the characteristics and intensity and frequency of cough in patients with IIPs, CTD-ILD, and CHP. Patients in the IIP group were more likely to be male and using an antifibrotic agent, and less likely to be using either glucocorticoid or proton pump inhibitor therapy than those in the CTD-ILD and CHP groups. The patients with CTD-ILD had a less extensive pack-year smoking history, higher FSSG scores, and better pulmonary function test results (i.e., FEV_1_, total lung capacity, and D_Lco_) than those with IIPs. The patients with CTD-ILD also had a greater total lung capacity than those with CHP. Among the three subtypes of ILD, the patients with the IIPs had the greatest intensity of cough but not frequency of cough. Patient age, current smoking, body mass index, MRC chronic dyspnoea scale, CPI, and LCQ scores were not affected by the subtype of ILD.

**Table 1:** Patient characteristics and intensity and frequency of cough

Variable	IIPs (*n* = 70)	CTD-ILD (*n* = 49)	CHP (*n* = 10)	*p*-value
Age, years, mean ± SD	69.4 ± 8.9	66.5 ± 11.4	71.3 ± 7.5	0.185
Male sex, n (%)	56 (80)*	13 (27)	7 (70)	< 0.001
Current smoker, n (%)	9 (13)	1 (2)	0 (0)	0.082
Pack-years of smoking, median (IQR)	40 (16–52)	0 (0–15)^†^	16 (4–35)	< 0.001
Body mass index^a^, mean ± SD	24.1 ± 3.3	23.1 ± 3.6	23.2 ± 2.0	0.279
FSSG score, median (IQR)	3 (1–7)	6 (2–12)^†^	4 (1–4)	0.010
MRC chronic dyspnoea scale, median (IQR)^b^	1 (1–2)	1 (1–2)	1 (1–3)	0.824
Pulmonary function tests, % predicted, median (IQR)^c^				
FEV_1_	82.9 (74.3–94.4)	94.2 (85.6–107.8)^†^	87.6 (78.8–90.1)	0.012
FVC	85.2 (74.2–97.3)	93.6 (80.2–106.1)	73.6 (68.8–93.3)	0.090
TLC	70.1 (64.3–85.8)	97.1 (79.8–104.1)^†‡^	68.5 (54.4–72.1)	< 0.001
D_LCO_	49.2 (38.8–58.9)	55.2 (48.1–66.6)^†^	56.0 (37.2–62.9)	0.024
Composite Physiologic Index, median (IQR)^c^	43.2 (30.5–52.8)	34.8 (31.1–47.5)	43.5 (34.5–51.2)	0.157
Medication, n (%)				
Glucocorticoid	9 (13)*	30 (61)	4 (40)	< 0.001
Antifibrotic agent	21 (30)*	0 (0)	2 (20)	< 0.001
Proton pump inhibitor	21 (30)*	26 (53)	4 (40)	0.040
VAS score for cough, mm, median (IQR)				
Intensity	31 (17–55)^§^	24 (8–46)	18 (6–20)	0.048
Frequency	24 (10–46)	13 (5–30)	10 (3–18)	0.060
Leicester Cough Questionnaire score, median (IQR)				
Physical	6.1 (5.5–6.4)	6.0 (5.0–6.5)	6.3 (6.0–6.6)	0.311
Psychological	6.6 (5.9–7.0)	6.3 (5.3–7.0)	6.8 (6.1–7.0)	0.378
Social	6.8 (6.0–7.0)	6.5 (5.3–7.0)	6.9 (6.0–7.0)	0.539
Total	19.3 (17.5–20.4)	18.7 (15.4–20.5)	19.6 (18.3–20.6)	0.447

The *p*-values are shown for across-group comparisons using one-way analysis of variance, the Kruskal-Wallis test, the chi-square test, or Fisher’s exact test as appropriate. Using a nominal 5% level of statistical significance, multiple comparisons were made using a Bonferroni-corrected significance level of 1.6% (*p* < 0.016). CHP, chronic hypersensitivity pneumonia; CTD-ILD, connective tissue disease-associated interstitial lung disease; D_Lco_, diffusing capacity of the lung for carbon monoxide; FEV_1_, forced expiratory volume in 1 s; FSSG, frequency scale for symptoms of gastro-oesophageal reflux disease; FVC, forced vital capacity; IIPs, idiopathic interstitial pneumonias; IQR, interquartile range; MRC, Medical Research Council; SD, standard deviation; TLC, total lung capacity; VAS, visual analogue scale. ^a^IIPs (*n* = 70), CTD-ILD (*n* = 49), CHP (*n* = 9); ^b^IIPs (*n* = 69), CTD-ILD (n = 49), CHP (*n* = 10); ^c^IIPs (*n* = 66), CTD-ILD (*n* = 48), CHP (*n* = 9). *p < 0.05 vs CTD-ILD or CHP; ^†^*p* < 0.016 vs IIPs; ^‡^*p* < 0.016 vs CHP; ^§^*p* = 0.067 vs CTD-ILD, *p* = 0.043 vs CHP

### Factors associated with cough intensity and frequency

**Fig. 1:**
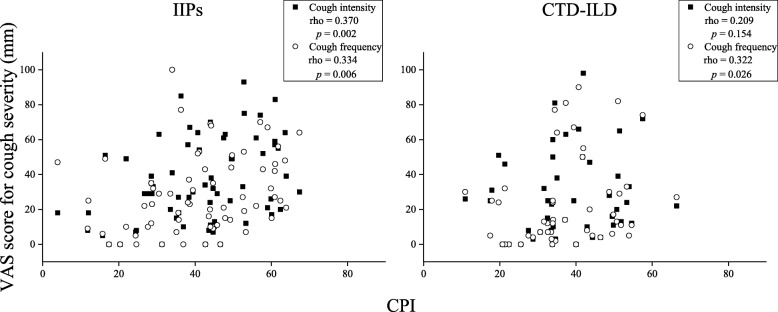
Association of intensity and frequency of cough with CPI. A statistically significant association was observed between intensity and frequency of cough and CPI in patients with idiopathic interstitial pneumonias. However, in connective tissue disease-associated interstitial lung disease, only the frequency of cough correlated with CPI. CPI, Composite Physiologic Index; CTD-ILD, connective tissue disease-associated interstitial lung disease; IIPs, idiopathic interstitial pneumonias; VAS, visual analogue scale

**Fig. 2:**
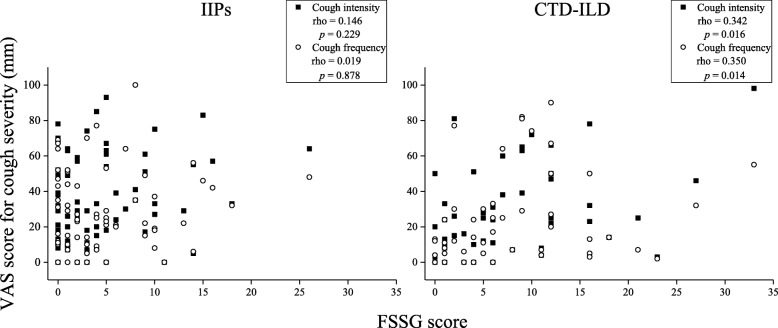
Association of intensity and frequency of cough with the FSSG score. A statistically significant association was observed between cough intensity and frequency and the FSSG score in patients with connective tissue disease-associated interstitial lung disease, but not in idiopathic interstitial pneumonias. CTD-ILD, connective tissue disease-associated interstitial lung disease; FSSG, frequency scale for symptoms of gastro-oesophageal reflux disease; IIPs, idiopathic interstitial pneumonias; VAS, visual analogue scale

The unadjusted factors associated with intensity and frequency of cough in ILD are shown in Tables 2 and 3, respectively. In patients with IIPs, both the intensity and frequency of cough were negatively associated with the D_Lco_ and positively with the CPI. For patients with CTD-ILD, the D_Lco_ and CPI were associated with cough frequency, and both the intensity and frequency of cough were associated with FSSG and the MRC chronic dyspnoea scale score. However, the association between cough frequency and either D_Lco_ or MRC chronic dyspnoea scale score was rather weak (Spearman rho = − 0.287 for D_Lco_ and rho = 0.291 for the MRC chronic dyspnoea scale score) in patients with CTD-ILD. Of note is that the association of cough intensity and frequency with CPI (Fig. 1) and FSSG score (Fig. 2) was numerically weaker and stronger, respectively, in patients with CTD-ILD than in those with IIPs. There was no significant association of either the intensity or frequency of cough with any variable in patients with CHP.

**Table 2:** Unadjusted analysis of factors associated with cough intensity in patients with interstitial lung disease

Variable	IIPs (*n* = 70)		CTD-ILD (*n* = 49)		CHP (*n* = 10)		Combined	
	ρ	*p*-value	ρ	*p*-value	ρ	*p*-value	ρ	*p*-value
Age, years	−0.065	0.595	0.063	0.669	0.339	0.337	−0.003	0.973
Male sex	–	0.644	–	0.658	–	0.833	–	0.737
Current smoker	–	0.598	–	0.490	–	–	–	0.156
Pack-years of smoking	−0.128	0.293	−0.144	0.323	−0.174	0.630	−0.031	0.728
Body mass index^a^	−0.297	0.013	−0.133	0.362	−0.025	0.949	−0.166	0.061
FSSG score	0.146	0.229	0.342	0.016	0.366	0.298	0.207	0.019
MRC chronic dyspnoea scale^b^	0.154	0.208	0.325	0.023	0.007	0.986	0.208	0.018
Pulmonary function tests, % predicted^c^								
FEV_1_	−0.118	0.344	0.021	0.887	0.097	0.805	−0.091	0.316
FVC	−0.206	0.097	−0.068	0.647	0.084	0.831	−0.123	0.175
TLC	−0.212	0.088	−0.011	0.943	−0.109	0.781	−0.186	0.040
D_LCO_	−0.316	0.010	−0.223	0.128	−0.310	0.417	−0.311	< 0.001
Composite Physiologic Index^c^	0.370	0.002	0.209	0.154	0.142	0.715	0.295	0.001
Medication								
Glucocorticoid	–	0.986	–	0.681	–	0.114	–	0.168
Antifibrotic agent	–	0.590	–	–	–	0.431	–	0.114
Proton pump inhibitor	–	0.156	–	0.074	–	0.830	–	0.057

The Spearman rank correlation was used to assess correlations between the data. The *p*-values for binary variables were determined using the Mann-Whitney *U* test. CHP, chronic hypersensitivity pneumonia; CTD-ILD, connective tissue disease-associated interstitial lung disease; D_Lco_, diffusing capacity of the lung for carbon monoxide; FEV_1_, forced expiratory volume in 1 s; FSSG, frequency scale for the symptoms of gastro-oesophageal reflux disease; FVC, forced vital capacity; IIPs, idiopathic interstitial pneumonias; MRC, Medical Research Council; TLC, total lung capacity. ^a^IIPs (n = 70), CTD-ILD (n = 49), CHP (n = 9); ^b^IIPs (n = 69), CTD-ILD (n = 49), CHP (n = 10); ^c^IIPs (n = 66), CTD-ILD (n = 48), CHP (n = 9)

**Table 3:** Unadjusted analysis of factors associated with cough frequency in patients with interstitial lung disease

Variable	IIPs (n = 70)		CTD-ILD (n = 49)		CHP (n = 10)		Combined	
	ρ	*p*-value	ρ	*p*-value	ρ	*p*-value	ρ	*p*-value
Age, years	0.017	0.890	0.222	0.125	−0.031	0.933	0.100	0.260
Male sex	–	0.174	–	0.441	–	0.667	–	0.415
Current smoker	–	0.079	–	0.857	–	–	–	0.037
Pack-years of smoking	−0.013	0.916	−0.192	0.186	−0.107	0.769	−0.011	0.898
Body mass index^a^	−0.251	0.036	−0.174	0.232	0.326	0.391	−0.166	0.061
FSSG score	0.019	0.878	0.350	0.014	0.342	0.334	0.174	0.049
MRC chronic dyspnoea scale^b^	0.071	0.561	0.291	0.042	0.356	0.312	0.173	0.051
Pulmonary function tests, % predicted^c^								
FEV_1_	−0.083	0.508	−0.040	0.786	0.038	0.923	−0.098	0.279
FVC	−0.160	0.201	−0.114	0.439	0.017	0.966	−0.120	0.187
TLC	−0.236	0.056	0.028	0.849	−0.126	0.748	−0.172	0.057
D_LCO_	−0.307	0.012	−0.287	0.048	−0.510	0.160	−0.328	< 0.001
Composite Physiologic Index^c^	0.334	0.006	0.322	0.026	0.259	0.500	0.319	< 0.001
Medication								
Glucocorticoid	–	0.642	–	0.096	–	0.257	–	0.012
Antifibrotic agent	–	0.362	–	–	–	0.066	–	0.049
Proton pump inhibitor	–	0.847	–	0.241	–	0.915	–	0.582

The Spearman rank correlation was used to assess correlations between the data. The *p*-values for binary variables were determined using the Mann-Whitney *U* test. CHP, chronic hypersensitivity pneumonia; CTD-ILD, connective tissue disease-associated interstitial lung disease; D_Lco_, diffusing capacity of the lung for carbon monoxide; FEV_1_, forced expiratory volume in 1 s; FSSG, frequency scale for the symptoms of gastro-oesophageal reflux disease; FVC, forced vital capacity; IIPs, idiopathic interstitial pneumonias; MRC, Medical Research Council; TLC, total lung capacity. ^a^IIPs (n = 70), CTD-ILD (n = 49), CHP (n = 9); ^b^IIPs (n = 69), CTD-ILD (n = 49), CHP (n = 10); ^c^IIPs (n = 66), CTD-ILD (n = 48), CHP (n = 9)

Table 4 shows the results of the multiple logistic regression analysis of factors associated with the intensity and frequency of cough in all patients with ILD. IIPs and the FSSG score were independently associated with both the intensity and frequency of cough. Furthermore, CPI tended to be independently associated only with the frequency of cough (*p* = 0.052).

**Table 4:** Multivariable analysis of factors associated with cough intensity and frequency in interstitial lung disease

Variable	Intensity^c^				Frequency^c^			
	AOR^a^	95% CI		*p*-value	AOR^a^	95% CI		*p*-value
		Lower	Upper			Lower	Upper	
Model (*n* = 122)^a^								
IIPs^b^	3.727	1.689	8.223	0.001	3.166	1.422	7.050	0.005
FSSG score	1.083	1.013	1.157	0.020	1.086	1.014	1.162	0.018
Composite Physiologic Index	–	–	–	–	1.029	1.000	1.060	0.052

AOR, adjusted odds ratio; CI, confidence interval; FSSG, frequency scale for symptoms of gastro-oesophageal reflux disease; IIPs, idiopathic interstitial pneumonias. ^a^Hosmer and Lemeshow test for cough intensity and frequency, *p* = 0.175 and *p* = 0.972, respectively. ^b^Connective tissue disease-associated interstitial lung disease or chronic hypersensitivity pneumonia as reference category. ^c^The data for intensity and frequency of cough were divided at the median to create categorical variables

### Features of the differences between cough intensity and frequency

Finally, we examined the features of the differences between cough intensity and frequency in all patients with ILD (*n* = 129). There was a positive correlation between cough intensity and cough frequency (Spearman rho = 0.801, *p* < 0.001), but notably, the cough frequency-dominant group had significantly lower total and subdomain LCQ scores than either the equal cough severity group or cough intensity-dominant group (Table 5). The Spearman’s correlation coefficients between total LCQ scores and intensity and frequency of cough were − 0.675 and − 0.762, respectively (both *p* < 0.001).

**Table 5:** Features of differences between cough intensity and frequency

	Cough frequency-dominant group	Equal cough severity group	Cough intensity-dominant group	*p*-value
	(*n* = 12)	(*n* = 79)	(*n* = 38)	
Type of interstitial lung disease, n (%)				0.858
IIPs	7 (58)	41 (52)	22 (58)	
CTD-ILD	5 (42)	30 (38)	14 (37)	
CHP	0	8 (10)	2 (5)	
Age, years, median (IQR)	73 (67–79)	70 (65–75)	65 (59–74)	0.048
Male sex, n (%)	7 (58)	47 (56)	22 (58)	0.986
Current smoker, n (%)	3 (25)	4 (5)	3 (8)	0.091
Pack-years of smoking, median (IQR)	13 (0–53)	22 (0–44)	20 (2–40)	0.925
Body mass index^a^, mean ± SD	22.7 ± 3.8	24.1 ± 3.3	23.1 ± 3.2	0.162
FSSG score, median (IQR)	8 (0–10)	3 (1–6)	5 (2–12)	0.158
MRC chronic dyspnoea scale, median (IQR)^a^	2 (2–3)	1 (1–2)	1 (1–3)	0.036
Pulmonary function tests, % predicted, mean ± SD^b^				
FEV_1_	87.2 ± 20.6	89.2 ± 19.1	87.7 ± 21.5	0.903
FVC	88.0 ± 24.3	89.8 ± 20.4	86.0 ± 20.4	0.664
TLC	85.0 ± 24.0	83.2 ± 21.8	78.1 ± 22.8	0.476
D_LCO_	50.9 ± 13.7	54.3 ± 15.5	50.7 ± 15.7	0.464
Composite Physiologic Index, mean ± SD^b^	40.9 ± 16.2	38.8 ± 13.6	42.3 ± 13.1	0.454
Medication, n (%)				
Glucocorticoid	1 (8)	27 (34)	15 (40)	0.132
Antifibrotic agent	2 (17)	13 (17)	8 (21)	0.826
Proton pump inhibitor	4 (33)	28 (35)	19 (50)	0.288
VAS score for cough, mm, median (IQR)				
Intensity	38 (26–45)*	15 (7–30)	54 (32–64)*	< 0.001
Frequency	67 (64–82)*^†^	14 (5–30)	21 (10–32)	< 0.001
Leicester Cough Questionnaire score, median (IQR)				
Physical	4.8 (3.4–5.7)*^†^	6.3 (5.9–6.6)	5.9 (5.3–6.4)*	< 0.001
Psychological	4.9 (3.9–6.4)*	6.7 (6.0–7.0)	6.3 (5.7–6.9)	0.002
Social	4.9 (3.9–6.1)*^†^	7.0 (6.0–7.0)	6.6 (5.5–7.0)	0.001
Total	13.9 (11.1–18.2)*^†^	19.6 (18.0–20.6)	18.7 (17.1–20.1)	< 0.001

To explore the features associated with a differences between the VAS scores for cough intensity and cough frequency, we stratified patients into three categories using the following formula: (cough intensity) − (cough frequency), defining a cough frequency-dominant group, ≤10 mm; an equal cough severity group, −9 mm to 9 mm; and a cough intensity-dominant group, ≥10 mm. *p*-values are shown for across-group comparisons using one-way analysis of variance, the Kruskal-Wallis test, chi-square test, or Fisher’s exact test as appropriate. Using a nominal 5% level of statistical significance, multiple comparisons were made using a Bonferroni-corrected significance level of 1.6% (*p* < 0.016). CHP, chronic hypersensitivity pneumonia; CTD-ILD, connective tissue disease-associated interstitial lung disease; D_Lco_, diffusing capacity of the lung for carbon monoxide; FEV_1_, forced expiratory volume in 1 s; FSSG, frequency scale for symptoms of gastro-oesophageal reflux disease; FVC, forced vital capacity; IIPs, idiopathic interstitial pneumonias; IQR, interquartile range; MRC, Medical Research Council; SD, standard deviation; TLC, total lung capacity; VAS, visual analogue scale. ^a^Cough frequency-dominant group (n = 12), equal cough severity group (*n* = 78), cough intensity-dominant group (n = 38); ^b^Cough frequency-dominant group (*n* = 11), equal cough severity group (*n* = 77), cough intensity-dominant group (*n* = 35). *p < 0.016 vs equal cough severity group; ^†^*p* < 0.016 vs cough intensity-dominant group

## Discussion

To the best of our knowledge, this is the first study to examine cough intensity and frequency separately in patients with IIPs, CTD-ILD, or CHP. Cough intensity was greatest in the group containing patients with IIPs; both the intensity and frequency of cough were negatively associated with the D_Lco_ and positively with the CPI in these patients. In patients with CTD-ILD, both components of cough correlated significantly with a higher FSSG score. In all patients with ILD, multiple logistic regression analysis revealed independent associations of IIPs and the FSSG score with both the intensity and frequency of cough, and a tendency for an independent association of CPI with the frequency of cough. Furthermore, although cough intensity and frequency behaved similarly overall, the total and subdomain LCQ scores were significantly poorer in the cough frequency-dominant group than in the other two groups.

Cheng et al. recently reported that the VAS score for cough severity was higher in patients with either IPF or CHP than in those with CTD (SSc)-ILD [5]. In the present study, we demonstrated that patients with IIPs had more intense cough than those with either CTD-ILD or CHP (Table 1), and that IIPs but not the CPI was an independent risk factor for the intensity of cough (Table 4). A VAS was the tool most frequently used in other studies to assess cough severity in patients with ILD [5] [8]. In those studies, severity was not usually defined clearly, but was assumed to relate to both the frequency and intensity of cough [19]. Although cough severity is not equivalent to cough intensity, the median score for cough intensity in the patients with IIPs in the present study (31 mm) was comparable with that for cough severity in patients with IPF reported on by Cheng et al. (39 mm) [5] and Key et al. (32 mm) [8].

In this study, CPI tended to be independently associated with the frequency of cough in patients with ILD. Specifically, in patients with IIPs, both the intensity and frequency of cough were associated with the CPI and D_Lco_, but not with FVC. We believe that no significant correlation was found between FVC and either the intensity or frequency of cough because of the maintenance of FVC in patients with ILD in this study. The CPI has been reported to correlate more strongly than the individual pulmonary function tests with the extent of disease seen on computed tomography scans in patients with IPF [26]. Therefore, it is possible that the severity of cough may be associated with the extent of the parenchymal lesions in IIPs. When compared with patients with CTD-ILD, there was also a numerically stronger association of both the intensity and frequency of cough with CPI in patients with IIPs (Fig. 1). Like our findings in patients with IIPs, Cheng et al. reported a significant correlation of cough severity with D_Lco_ in patients with IPF [5]. Although our results do not indicate a causal relationship between the intensity and frequency of cough and CPI, Ryerson et al. showed that cough in patients with IPF is an independent predictor of disease progression, which was defined as a 15% decline in D_Lco_, a 10% decline in FVC, or lung transplantation or death attributable to any cause [3]. Moreover, it has been suggested that the fibroblastic foci that develop in IPF may result from stretch injury at the epithelial-mesenchymal interface [27]. It was also found that both the intensity and frequency of mechanical stress-related breathing resulted in activation of transforming growth factor beta-1 in rat fibrotic tissue; when forces of 5–20 mN were applied to fibrotic lung strips, active transforming growth factor beta-1 increased significantly in response to the mechanical stimulus [13]. Therefore, an increase in the intensity and frequency of cough that causes mechanical stress might contribute to disease progression, such as an increase in CPI, in patients with IIPs.

Alternatively, cough in patients with IIPs might be a consequence of architectural distortion of the fibrotic lung. Traction bronchiectasis is caused by constriction of the surrounding fibrotic alveolar tissue, and such architectural distortion of the bronchial tree may be involved in the activation of rapidly adapting receptors [10, 28], resulting in an exaggerated coughing response, i.e., an increase in either cough intensity or frequency. Further studies are required to evaluate the correlation between the extent of disease seen on HRCT and the intensity and frequency of cough in patients with IIPs.

The FSSG score was another independent risk factor for both the frequency and intensity of cough in patients with ILD, particularly in patients with CTD-ILD. This finding is consistent with that of Tashkin et al., suggesting that the frequency of cough relates to the severity of GORD at baseline and declines in parallel with an improvement in GORD by treatment for SSc-ILD [11]. Furthermore, a study in patients with SSc-ILD reported a relationship between the degree of pulmonary fibrosis assessed using a validated HRCT score and the number of reflux episodes in the distal and proximal oesophagus [29]. Hence, the well-known interaction between GORD and cough [30] may be closely involved in the pathogenesis of CTD-ILD, particularly SSc-ILD. Meanwhile, FSSG scores were lower in patients with IIPs than those in patients with CTD-ILD, and there was no significant correlation of either intensity or frequency of cough with the FSSG score in patients with IIPs in the present study. Another study found that only 25% of patients with IPF and increased exposure to acid in the oesophagus reported typical reflux symptoms [31]. A further study found no association between GORD and cough in patients with IPF [3]. Therefore, the impact and association of GORD with cough may be weaker in patients with IIPs than in those with CTD-ILD, even though GORD is thought to be involved in the pathogenesis of IIPs [32].

Patients with ILD and frequency-dominant cough had a greater impairment of health status than those in the other groups. Numerically, there was a stronger correlation between cough frequency and total LCQ scores than between cough intensity and total LCQ scores. Our results concur with a report by Key et al. that demonstrated a strong correlation between the objective cough count and cough-related HRQoL in patients with IPF [8]. Furthermore, given the tendency for an association between frequency of cough and the CPI in patients with ILD in the present study, it is reasonable to assess the frequency of cough in patients with ILD in a clinical setting. However, we should not neglect cough intensity. The physical domain scores of the LCQ were significantly poorer in the cough intensity-dominant group than in the group with equal cough severity (Table 5). Furthermore, the intensity of cough also showed a significant correlation with total LCQ scores in patients with ILD. Finally, in patients with IIPs, cough intensity, which was severest in the three subtypes of ILD, was as significantly associated with D_Lco_ and CPI as cough frequency. These findings highlight the need for the assessment of both the intensity and frequency of cough in patients with ILD, particularly for patients with IIPs.

The strengths of this study are that it addressed both the intensity and frequency of cough in patients with ILD and included an analysis of the subtypes of ILD (i.e., IIPs, CTD-ILD, and CHP). However, the study also has several limitations. First, the small numbers of patients with CHP may limit the generalisability of the results obtained. Second, we did not investigate whether or not patients had sputum present*.* Third, the study had a cross-sectional design that precluded the identification of either a temporal or causal relationship. Prospective and longitudinal studies that include additional characteristics of cough (e.g., production of sputum and duration) are needed*.* Fourth, the correlation coefficients in the bivariate analysis were low. Therefore, the relationship between cough intensity and frequency and clinical indices identified in this study requires further investigation. Fifth, there was a risk of loss of statistical power when continuous variables were dichotomised for multivariate analysis. However, dichotomisation was the best way of performing a multivariate analysis to identify independent predictors of cough intensity and frequency in this study because the raw data and log-transformed data for these two variables were not normally distributed. Finally, we only used subjective tools to assess the intensity and frequency of cough. Further research that includes the use of both subjective and objective tools is needed.

## Conclusions

We found that the degrees of patient-reported cough intensity and frequency and their associations with clinical indices representing disease severity were different for each type of ILD. We also found independent associations of IIPs and the FSSG score with both the intensity and frequency of cough*,* and a tendency for an independent association of the CPI with the frequency of cough in patients with ILD. Furthermore, impairment of cough-specific HRQoL was noted in the cough frequency-dominant group. These findings suggest that medical staff could manage patients with ILD by considering cough-related factors when assessing the intensity and frequency of cough.

## Data Availability

The datasets generated and/or analysed during the current study are not publicly available due to limitations on secondary use but are available from the corresponding author on reasonable request.

## Abbreviations

CHP: Chronic hypersensitivity pneumonia
CPI: Composite Physiologic Index
CTD-ILD: Connective tissue disease-associated interstitial lung disease
D_Lco_: Diffusing capacity of the lung for carbon monoxide
FEV_1_: Forced expiratory volume in 1 s
FSSG: Frequency scale for symptoms of gastro-oesophageal reflux disease
FVC: Forced vital capacity
GORD: Gastro-oesophageal reflux disease
HRCT: High-resolution computed tomography
HRQoL: Health-related quality of life
IIPs: Idiopathic interstitial pneumonias
ILD: Interstitial lung disease
IPF: Idiopathic pulmonary fibrosis
LCQ: Leicester Cough Questionnaire
MRC: Medical Research Council
SSc: Systemic sclerosis
VAS: Visual analogue scale
